# Socioeconomic position and body composition across the life course: a systematic review protocol

**DOI:** 10.1186/s13643-019-1197-z

**Published:** 2019-11-07

**Authors:** Charis Bridger Staatz, Yvonne Kelly, Rebecca Lacey, Rebecca Hardy

**Affiliations:** 1 MRC Unit for Lifelong and Health Ageing at UCL, Department of Population Health and Experimental Medicine, University College London, London, UK; 2 Department of Epidemiology and Public Health, University College London, 1-19 Torrington Place, London, WC1E 7HB UK

**Keywords:** Body composition, Socioeconomic position, Fat mass, Lean mass, Fat-free mass, Life course

## Abstract

**Introduction:**

The relation between socioeconomic position (SEP) and obesity measured by body mass index (BMI) has been extensively reviewed, but there is less research on the association between SEP and body composition. Fat distribution and muscle quality have been linked to adverse health outcomes such as cardiovascular disease, diabetes and poor physical capability. There is some evidence of secular changes in body composition with increasing fat-mass and reducing muscle quantity and strength, but it is unclear whether there have been secular changes in social inequalities in body composition. The aim is to perform a systematic review of the existing literature on the association between SEP and body composition and to explore any secular changes.

**Methods:**

The systematic review will be carried out according to PRISMA guidelines. An electronic search of MEDLINE and Embase Classic + Embase will be conducted using OvidSP as the database interface, as well as SPORTDiscus using EBSCO. Two independently working reviewers will initially screen abstracts to exclude papers that are clearly ineligible, followed by a full-text screening to exclude papers not meeting all inclusion criteria. Any disagreements will be resolved through discussion. Data extraction and quality assessment of eligible papers will be carried out by 2 reviewers using a standardised form. The reference lists of identified papers will be searched for additional papers. Original studies in the English language, which examine the association between SEP at any age and body composition at the same or later age will be included if they use any recognised measures of SEP (e.g. income, occupation, over-crowding) and a recognised measure of body composition (total, proportional or location of fat mass and fat-free mass, using any appropriate methods, excluding anthropometry). Due to expected heterogeneity, a narrative synthesis is expected, with a descriptive summary to be provided in tables. If there is consistency in reporting of associations, a random-effects meta-analysis will be used to provide an overall summary estimate.

**Discussion:**

The results of the review will summarise the existing evidence on social inequalities in body composition. Findings will identify gaps in knowledge and where further research is required.

**Systematic review registration:**

PROSPERO CRD42019119937

## Background

Obesity has repeatedly been linked to socioeconomic position (SEP) in industrialised countries, with the association being the subject of multiple systematic reviews [1–6]. These reviews demonstrate a predominantly inverse relationship between SEP and obesity measured by anthropometric measures such as body mass index (BMI) among all age groups, and with associations tending to be stronger among women compared to men [3, 4, 6, 7]. There is evidence that associations differ dependent on the SEP measure used, with stronger associations between SEP and obesity in both men and women when measured by education [1, 2, 8]. Furthermore, in light of secular increases in obesity—with those born post-1980 having a three-fold higher likelihood of being overweight and obese [9]—there is evidence that the inequalities in overweight and obesity are increasing [7] with these increase particularly evident across childhood [10] and at the upper end of the BMI distribution [11].

Although the literature linking SEP and BMI has been extensively reviewed, the smaller number of studies linking SEP and body composition have not. The majority of evidence on inequalities in overweight and obesity come from studies using BMI, a measure of weight for height which does not distinguish fat from lean mass. Measures of body composition provide an estimate of the proportion of fat mass to fat-free mass, including lean mass, and can inform about the location of fat mass [12]. A higher proportion of fat-to-lean mass has been shown to be important in the risk of cardiovascular disease [13]. Both total and proportion of fat mass has been associated with cardiovascular and metabolic disease, with higher central adiposity and android-to-gynoid fat mass ratio implicated in increased risk [14–17]. In addition lean mass plays a role in the development of insulin sensitivity, with muscle tissue being a sight of glucose uptake, therefore having the potential to impact the onset of diabetes and other metabolic conditions [18, 19].

If inequalities in body composition, and in particular fat mass, are similar or stronger than the inequalities in BMI, this has major public health implications as the impact of inequalities in adiposity on health may have been underestimated when adiposity is based on BMI. Further, evidence from serial data indicates secular changes in body composition among children, with a significant decline in muscle fitness when adjusted for height and weight observed in 10-year-old children between 1998 and 2014 in the UK [20] and positive secular trends for fat mass index from 1960 to 1999 in the USA [21]. If there are secular changes in muscle and fat acquisition in childhood, this may lead to detrimental secular changes in adult body composition because as people age, BMI increases more likely reflect fat acquisition than muscle [22].

We therefore aim to carry out a systematic review of the literature to assess the association between SEP and measures of body composition (in particular fat mass, lean mass and the location of fat mass) in the general population, comparing differences in body composition between those of high SEP against those of lower SEP. We will assess associations between (a) SEP and body composition in childhood (up to and including 18 years), (b) SEP and body composition in adulthood and (c) childhood SEP and adult body composition. We also aim to assess whether the socioeconomic inequalities in body composition have increased in more recent cohorts.

## Methods

This protocol has been registered with the PROSPERO database (PROSPERO CRD42019119937) and has been reported using the Preferred Reporting Items for Systematic Reviews and Meta-Analyses Protocols (PRISMA-P) checklist (Additional file 1). Any important amendments to the protocol will be made through updating the PROSPERO record.

### Definition of key terms

#### (a) Socioeconomic position

Socioeconomic position (SEP) to be measured by any recognised indicators of social position in society, e.g. income, education, overcrowding, area-level deprivation [23]. The same indicators of parental SEP will be used as a marker for childhood SEP.

#### (b) Measures of body composition

Body composition will be defined as any measurement related to total fat mass and fat-free mass, location of fat mass and fat-free mass or any proportion or ratio of measures of fat mass and fat-free mass. Body composition will be measured using any appropriate measure, excluding anthropometric indicators. Appropriate methods of measurement are listed below [12, 24]:

i) Bioelectrical impedance analysis (BIA)
ii) Dual X-ray absorptiometry (DXA)
iii) Computed tomography (CT)
iv) Magnetic resonance imaging (MRI)
v) Other less common methods: total body water (TBW), bone density or densitometry, total body counting and neutron activation and air-displacement plethysmography

### Eligibility criteria

Studies will be included if they:

- Are original studies published in peer-reviewed journals
- Examine the association between at least one measure of SEP and a measure of body composition at the same or a later age
- Use any recognised measure of SEP as described above
- Use any recognised measure of body composition as described above
- Are an observational study, such as prospective and retrospective cohorts, cross-sectional and case-control studies
- Use samples selected from the general population
- Are written in the English language

Studies will be excluded if they:

- Do not meet inclusion criteria
- Are reviews
- Studies in specific groups, e.g. clinical or patient populations
- Measure body composition through anthropometric measures, such as BMI, waist circumference, waist-hip ratio and waist-height ratio

### Search strategy

**Fig. 1:**
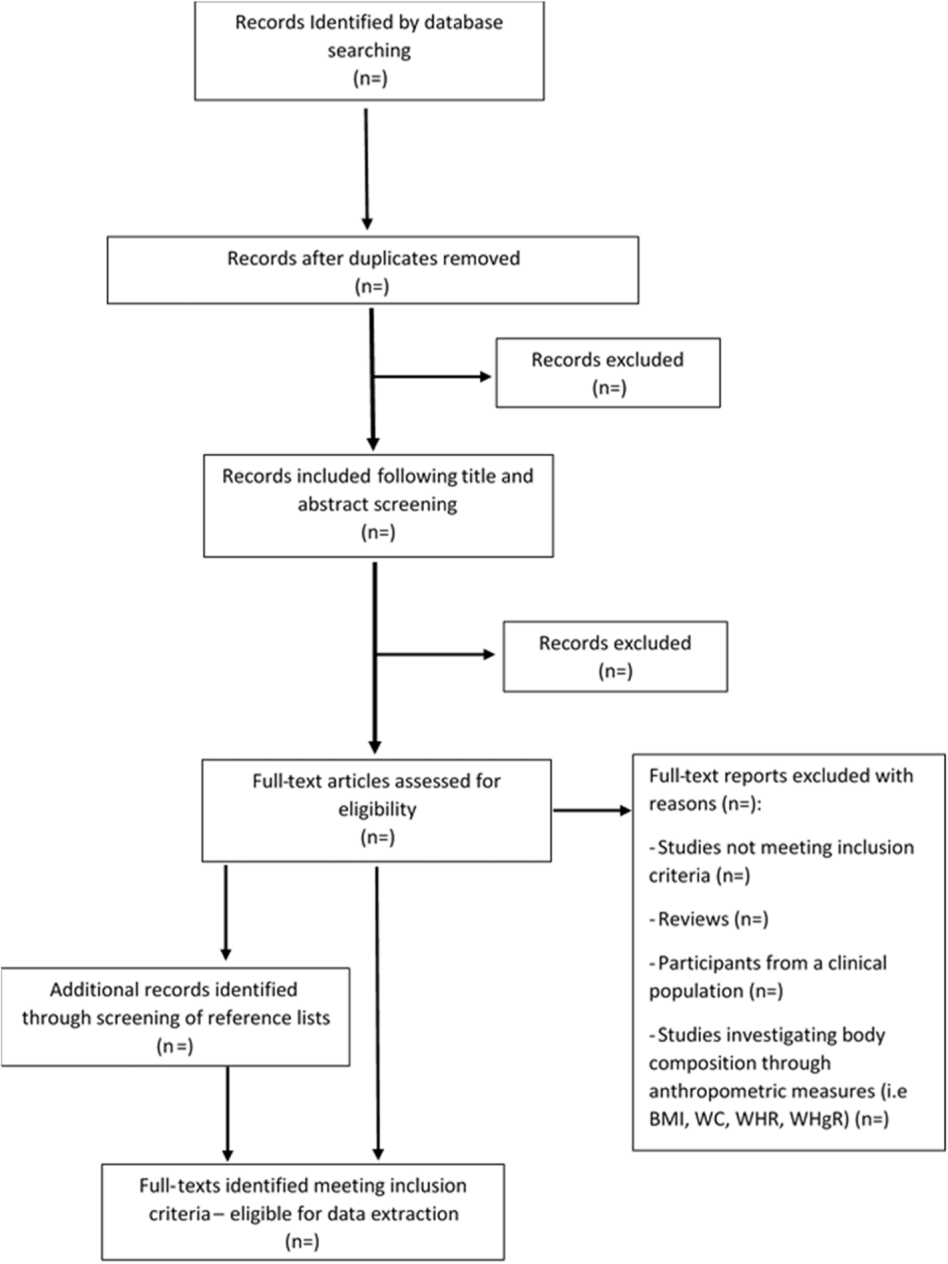
PRIMSA flow diagram

An electronic search will be carried out to identify appropriate studies, with MEDLINE and Embase Classic + Embase being searched using OvidSP as the interface, as well as a search of SPORTDiscus using EBSCO as the interface. Databases will be searched from the earliest record entry until 30th of January 2019. Search terms are detailed in Table 1. Different tools and techniques will be adopted to ensure the search identifies all relevant articles, as documented in Table 2. The reference list of eligible full texts will also be screened to identify further additional articles (Fig. 1).

**Table 1:** Search terms

SEARCH TERMS	
Database	MeSH terms
Medline	Body Composition – exp. Body Composition/; Adipose Tissue/; exp. Body Fat Distribution/; Obesity/or obesity, abdominal/.
	Body Composition Measures - Electric Impedance/; Magnetic Resonance Imaging/; Tomography, X-Ray Computed/; Densitometry/; Whole-Body Counting/; Plethysmography/.
	Socioeconomic Position - socioeconomic factors/ or poverty/ or poverty areas/ or social class/; Educational status/ or income/ or occupations/ or social conditions/.
Embase + Embase Classic	Body Composition - Body composition/ or body distribution/ or body fat/ or body fat distribution/; Obesity/; lean body weight/; Fat mass/.
	Body Composition Measures - Impedance/; nuclear magnetic resonance imaging/; computer assisted tomography/; densitometry/; whole body counting/; Total body water/; plethysmography/.
	Socioeconomic Position - socioeconomics/ or educational status/ or income group/ or poverty/; income/ or occupation/ or household income/; social status/ or social background/ or social class/; education/;
SPORTDiscuss	Body Composition - ((DE "BODY composition" OR DE "HUMAN body composition") OR (DE "OBESITY")) OR (DE "ADIPOSE tissues")
	Body Composition Measures - ((((DE "BIOELECTRIC impedance") OR (DE "COMPUTED tomography")) OR (DE "MAGNETIC resonance imaging")) OR (DE "BONE densitometry")) OR (DE "PLETHYSMOGRAPHY")
	Socioeconomic Position - ((DE "EDUCATION") OR (DE "EDUCATIONAL attainment")) OR (DE "HEALTH & income")
Free-text search terms	
Body composition	1. Body Composition MeSH Terms
	2. (Body adj3 (composition or distribution))
	3. ((fat or adipos*) adj3 (composition or distribution or mass or index or kg or total))
	4. ((muscl* or lean) adj3 (composition or distribution or mass or index or kg or total))
	5. ((fat-free) adj3 (mass or kg or total))
	6. ((android or gynoid or visceral or appendicular or abdominal or intra-abdominal) adj3 (fat or lean or muscle or mass or adipos*))
	7. 1 OR 2 OR 3 OR 4 OR 5 OR 6
Body composition measures	8. Body Composition Measures MeSH Terms
	9. ((impedance) adj3 (bioelectrical or foot-to-foot or hand-to-foot or analy?is))
	10. (bioimpedance or body fat analy?er or body composition analy?er or tanita)
	11. (dual x-ray absorptiometry or DEXA or DXA or dual-energy X-ray absorptiometry)
	12. (magnetic resonance imaging or MRI)
	13. (Computed tomography or CT or CAT scan)
	14. (densitometry)
	15. ((neuron activation or total body counting or whole body counting))
	16. (total body water)
	17. (air-displacement plethysmography)
	18. 8 OR 9 OR 10 OR 11 OR 12 OR 13 OR 14 OR 15 OR 16 OR 17
	19. 7 AND 18
Socioeconomic position	20. Socioeconomic Position MeSH terms
	21. (social class or social status or social position or socio-economic or socioeconomic or social circumstance*)
	22. (sociodemo*)
	23. Occupation*
	24. Educat*
	25. (income* or manual or class)
	26. (depriv* or poverty or overcrowding)
	27. 20 OR 21 Or 22 OR 23 OR 24 OR 25 OR 26
	28. 19 AND 27
	29. Limit to English Language (and Human in OvidSP)

**Table 2:** Tools and techniques for searching databases

Technique and description	Command	Example
All known synonyms of key words		*socioeconomic position* may include *socio-economic*, *education*, *occupation*, *income* etc.
Replace up to one character in the word—allows alternative spellings to be included.	?	*Analy?er* Would include both *Analyser* and *Analyzer*
Truncation command—used to acknowledge and capture alternative endings to words.	“root word”*	Adipos* would additionally search for *adiposity* and *adipose*
Boolean logic operators—used to (a) identify results with at least one of the search terms present and (b) to combine results of different search terms.	a) “OR” b) “AND”	a) *Muscle OR Lean Mass Index* would retrieve articles that either have terms.
		b) *Body Composition AND Socioeconomic Position* would only retrieve articles with both terms.
Proximity operators—used to identify words within a specified distance of each other.	Ovid: adj3 ESBCO: n3	Occupation* adj3 father* would identify articles whereby “occupation” and “father” are within three words of each other.

### Study selection

Results of the search will be de-duplicated and stored in the reference manager, EndNote. This database will be exported to Rayyan QCRI [25] and two researchers will independently screen each title and abstract for eligibility. Full texts of the potentially eligible articles will be retrieved using the UCL findit@UCL linking service, and where difficult to access, authors will be contacted directly. Full texts will also be duplicated and each will be screened by two authors working independently. Reasons for exclusion will be recorded. Any disagreement regarding eligibility of the article will be resolved via discussion.

### Data extraction and quality assessment

The reviewers will extract the following relevant information: citation details including title and year of publication; study details including design of the study, country or region, sample size; exposure and outcome details including measurement method and time in life-course of data collection; details of participants including age, sex and year of birth; statistical methods used in analysis; any reported potential confounders and mediators. A data extraction form will be used (Additional file 2). All data will be extracted by 2 independent reviewers and discrepancies resolved through discussion.

Assessment of study quality will be carried out using an amended version of the Newcastle-Ottawa Quality Assessment scale [26] (Additional file 3).

### Synthesis

#### Narrative synthesis

A descriptive summary of the findings will be provided in tables, with studies being grouped into those that examine: (a) childhood SEP and body composition, (b) adult SEP and adult body composition and (c) childhood SEP and adult body composition. The narrative synthesis will follow the Economic and Social Research Council Methods Programme guidelines [27], with a focus on identifying and exploring sources of heterogeneity.

#### Meta-analysis

Given there is consistency in reporting of associations for any body composition measure, a random-effects meta-analysis will be used to pool the estimates from the studies to provide an overall summary estimate and results will be presented in a forest plot. The degree of heterogeneity will be assessed using Higgins Thompson *I*^2^ test and Cochran’s *Q* test, with publication bias being assessed through a funnel plot. Subgroup analysis or meta-regression will be carried out to assess sources of heterogeneity selected a priori: (i) birth cohort, (ii) sex and (iii) SEP measure.

#### Reporting

The findings of this systematic review will be reported in accordance with the Preferred Reporting Items for Systematic Reviews and Meta-Analyses (PRISMA) guidelines [28].

## Discussion

This study will systematically review the literature examining the link between SEP and body composition across the life course. The relationship between SEP measured in childhood and adulthood, and body composition measured at the same or later point will be explored, with other sources of heterogeneity being investigated. The strengths and limitations of the evidence will be considered, therefore assessing potential bias, and the findings of the review will be discussed in the context of related reviews. The results of the review will summarise the existing evidence on social inequalities in body composition and identify where there are gaps in knowledge were further research is required.

## Supplementary information

**Additional file 1.** PRISMA-P 2015 Checklist.

**Additional file 2.** Data Extraction Form.

**Additional file 3.** Quality Assessment Form.

## Data Availability

Not applicable

## Abbreviations

BIA: Bioelectrical impedance analysis
BMI: Body mass index
CT: Computed tomography
DXA: Dual X-ray absorptiometry
MRI: Magnetic resonance imaging
PRISMA: Preferred Reporting Items for Systematic Reviews and Meta-Analyses
SEP: Socioeconomic position
