# Supplementary material for: Socioeconomic position and body composition across the life course: a systematic review protocol
Source: Syst Rev. 2019 Nov 7;8:263. doi: 10.1186/s13643-019-1197-z (PMC6836397; doi:10.1186/s13643-019-1197-z)
Supplement: Supplementary file 2 — Additional file 2. Data Extraction Form. [file 13643_2019_1197_MOESM2_ESM.docx]

# Additional File 2. Data Extraction Form

**Inclusion/Exclusion form:** Socioeconomic position and body composition

| Reference details |  |  |  |  |  |  |
| --- | --- | --- | --- | --- | --- | --- |
| A1. Ref ID |  | | | | | |
| A2. 1^st^ Author |  | | | | | |
| A3. Title of paper |  | | | | | |
| A4. Journal |  | | | | | |
| A5. Volume |  | | | | | |
| A6. Year of publication |  | | | | | |
| A7. Publication type | Paper^1^ |  | Abstract^2^ |  |  |  |
| A8. Assessor’s name | CBS^1^ |  | AG^2^ |  | JB^3^ |  |
| A9. Date |  | | | | | |

| Yes^1^ |  | No^2^ |  |
| --- | --- | --- | --- |

B. Study included in systematic review:

| Reason(s) for exclusion (if excluded): | Yes^1^ | No^2^ |
| --- | --- | --- |
| C1. Ineligible exposure |  |  |
| C2. Ineligible outcome |  |  |
| C3. Ineligible population (i.e clinical population) |  |  |
| C4. Review article |  |  |
| C5. Duplicate (Insert Ref ID of other study) |  |  |
|  | Ref ID: |  |
| C6. Not in English language |  |  |
| C7. Other please specify: |  |  |

**Data extraction form:** Socioeconomic position and body composition

| Reference details |  |  |  |  |  |  |
| --- | --- | --- | --- | --- | --- | --- |
| A1. Ref ID |  | | | | | |
| A2. 1^st^ Author |  | | | | | |
| A3. Title of paper |  | | | | | |
| A4. Journal |  | | | | | |
| A5. Volume |  | | | | | |
| A6. Year of publication |  | | | | | |
| A7. Publication type | Paper^1^ |  | Abstract^2^ |  | Other^3^ |  |
| A8. Assessor’s name | CBS^1^ |  | AG ^2^ |  | JB ^3^ |  |
| A9. Date |  | | | | | |

| Study details |  |  |  |  |  |  |  |  |  |  |  |  |
| --- | --- | --- | --- | --- | --- | --- | --- | --- | --- | --- | --- | --- |
| B1. Name of study/cohort |  | |  |  |  |  |  |  | |  |  |  |
| B2. Design | Cross-sectional^1^ | |  | Prospective cohort^2^ |  | Retrospective cohort^3^ |  | Case-control^4^ | |  | Other^5^ |  |
| B2A. If other: |  | | | | | | | | | | | |
| B2B. Participants birth year, if given |  | | | | | | | | | | | |
| B3. Country | US^1^ | |  | UK^2^ |  | AUS^3^ |  |  | Other^4^ | |  | |
| B3A. If other: |  |  | | | | | | | | | | |

| SEP exposures used |  |  |  |  |  |  |  |  |  |  |  |  |
| --- | --- | --- | --- | --- | --- | --- | --- | --- | --- | --- | --- | --- |
| C1. Own education | Yes^1^ | | | |  | | No^2^ | | | | |  |
| C2. Own Occupation | Yes^1^ | | | |  | | No^2^ | | | | |  |
| C3. Own Income | Yes^1^ | | | |  | | No^2^ | | | | |  |
| C.4 Own Social Class | Yes^1^ | | | |  | | No^2^ | | | | |  |
| C.5 Own SEP | Yes^1^ | | | |  | | No^2^ | | | | |  |
| C6. Father’s education | Yes^1^ | | | |  | | No^2^ | | | | |  |
| C7. Mother’s education | Yes^1^ | | | |  | | No^2^ | | | | |  |
| C8. Father’s occupation | Yes^1^ | | | |  | | No^2^ | | | | |  |
| C9. Mother’s occupation | Yes^1^ | | | |  | | No^2^ | | | | |  |
| C10. Father’s Social Class | Yes^1^ | | | |  | | No^2^ | | | | |  |
| C11. Mother’s Social Class | Yes^1^ | | | |  | | No^2^ | | | | |  |
| C12. Family Income | Yes^1^ | | | |  | | No^2^ | | | | |  |
| C13. Family SEP | Yes^1^ | | | |  | | No^2^ | | | | |  |
| C14. Area-level deprivation index | Yes^1^ | | | |  | | No^2^ | | | | |  |
| C15. Other please list: |  | | | | | | | | | | | |
| C16. How ascertained | Prospectively^1^ | | |  | | Retrospectively^2^ | | | | |  | |
| C17. Age recorded (yrs.) |  | | | | | | | | | | | |
| C18. Age Recorded (Grouped) | Childhood^1^ |  | Adolescence^2^ | | | | |  | Adulthood^3^ |  | | |

| Body Composition Outcomes | |  |  |  |  |  |  |  |  |  |  |  |  |  |  |  |
| --- | --- | --- | --- | --- | --- | --- | --- | --- | --- | --- | --- | --- | --- | --- | --- | --- |
| D1. Body Composition Measure | Fat Mass Index^1^ | |  | Lean Mass index^2^ |  | Proportion Fat-to-Lean^3^ | |  | Location of fat or fat free mass ^4^ | |  | | Other^5^ | |  | |
| D1A. If location please list |  | | | | | | | | | | | | | | | |
| D1B. If other please describe |  | | | | | | | | | | | | | | | |
| D2. How ascertained | BIA | |  | MRI |  | | DXA |  | | CT | |  | | Other | |  |
| D2B. If Other, please describe |  | | | | | | | | | | | | | | | |
| D4. Age(s) ascertained (yrs.) |  | | | | | | | | | | | | | | | |
| D5. Comments |  | | | | | | | | | | | | | | | |

| Available participant numbers |  |  |  |  |  |  |  |
| --- | --- | --- | --- | --- | --- | --- | --- |
| E1. Baseline | Yes^1^ |  | No^2^ | |  | If yes, number |  |
| E2. Excluded | Yes^1^ |  | No^2^ | |  | If yes, number |  |
| E3. Refused | Yes^1^ |  | No^2^ | |  | If yes, number |  |
| E4. Lost to follow-up | Yes^1^ |  | No^2^ | |  | If yes, number |  |
| E5. Other losses | Yes^1^ |  | No^2^ | |  | If yes, number |  |
| E6. Included in analysis | Yes^1^ |  | No^2^ |  | | If yes, number |  |
| E7. All accounted for | Yes^1^ |  | No^2^ |  | |  |  |

| Variable details |  |  |  |  |  |  |
| --- | --- | --- | --- | --- | --- | --- |
| F1. SEP Measure | Continuous^1^ |  | Categorical^2^ |  | Quantiles^3^ |  |
| F2. Body Composition | Categories ^1^ |  | Continuous (original scale)^2^ |  | Other ^3^ |  |
| F2A. If other please describe |  | | | | | |
| F3. Comments |  | | | | | |

| Analysis |  |  |  |  |  |  |  |
| --- | --- | --- | --- | --- | --- | --- | --- |
| G1. How results analysed | Linear regression^1^ |  | Logistic regression^2^ |  | Other^3^ | |  |
| G1A. If other: |  | | | | | | |
| G2. Included in analysis | Males and females^1^ |  | Males only^2^ |  | | Females only^3^ |  |
| G3. List models presented incl. unadjusted |  | | | | | | |
|  |  | | | | | | |

| Effect estimates |  |  |  |  |  |  |
| --- | --- | --- | --- | --- | --- | --- |
| Association tested and which group e.g. M/F/All | | No. analysed | Type of effect estimate and category comparison/value of unit change | Effect estimate | 95% CI; SE; p | Avai |
|  | |  |  |  |  |  |
|  | |  |  |  |  |  |
