# Supplementary material for: Socioeconomic position and body composition across the life course: a systematic review protocol
Source: Syst Rev. 2019 Nov 7;8:263. doi: 10.1186/s13643-019-1197-z (PMC6836397; doi:10.1186/s13643-019-1197-z)
Supplement: Supplementary file 3 — Additional file 3. Quality Assessment Form. [file 13643_2019_1197_MOESM3_ESM.docx]

## Additional File 3. Quality Assessment Form

**NEWCASTLE - OTTAWA QUALITY ASSESSMENT SCALE (amended)**

Note: A study can be awarded a maximum of one star for each numbered item within the Selection and Outcome categories. A maximum of two stars can be given for Comparability

**Selection**

1) Representativeness of the exposed cohort

a) Truly representative of the source population **🟑**

b) Somewhat representative of the source population**🟑**

c) Selected group of users e.g. nurses, volunteers

d) No description of the derivation of the cohort

2) Ascertainment of SEP

a) Prospectively from parents/family/own (or linking to area-level indicators) **🟑**

b) Structured interview (recall) **🟑**

c) Written self-report

d) No description

**Comparability**

1) Comparability of cohorts on the basis of the design or analysis

a) Study controls for birth weight **🟑**

b) Study controls for any additional relevant factors (e.g. age, sex, ethnicity) **🟑**

c) Only unadjusted model presented

**Outcome**

1) Assessment of body composition

a) Measure indexed to body size or ratio (i.e fat mass index or fat:lean ratio) **🟑**

b) No indexation or ratio

c) No description

2) Was follow-up long enough for outcomes to occur

a) Longitudinal **🟑**

b) Cross-sectional

3) Adequacy of follow up of cohorts

a) Complete follow up - all subjects accounted for **🟑**

b) Subjects lost to follow up unlikely to introduce bias - small number lost - > 75% follow up, or description provided of those lost) **🟑**

c) Follow up rate < 75% and no description of those lost

d) No statement

≥ 6 **🟑**= high quality

< 6 **🟑**= low quality
